# Endoplasmic reticulum stress is associated with cardiac effects of bisphenol S independently of a high-fat diet

**DOI:** 10.1007/s00418-026-02525-2

**Published:** 2026-08-03

**Authors:** Beatriz Alexandre-Santos, Guilherme dos Santos Reis, Luiza Mazzali Ferraz, Maria Eduarda Lima da Silva, Gabriel Ferreira Lima, Fernanda Carla Ferreira Brito, Milena Barcza Stockler-Pinto, Antonio Claudio Lucas da Nóbrega, Leandro Miranda-Alves, D’Angelo Carlo Magliano, Eliete Dalla Corte Frantz

**Affiliations:** 1 Department of Morphology, Laboratory of Exercise Sciences, Biomedical Institute, Fluminense Federal University, Rua Alameda Barros Terra, S/N, Sao Domingos, Niteroi, RJ 24.020-150 Brazil; 2 Research Center On Morphology and Metabolism, Biomedical Institute, Fluminense Federal University, Niteroi, RJ Brazil; 3 Laboratory of Experimental Pharmacology, Biomedical Institute, Fluminense Federal University, Niteroi, RJ Brazil; 4 Research Center On Nutrigenetics and Nutrigenomics, Faculty of Nutrition, Fluminense Federal University, Niteroi, RJ Brazil; 5 Laboratory of Experimental Endocrinology, Institute of Biomedical Science, Federal University of Rio de Janeiro, Rio de Janeiro, RJ Brazil; 6 National Institute of Endocrine Disruptors and Endocrine-Metabolic Repercussions, Rio de Janeiro, Brazil

**Keywords:** Bisphenol S, Obesity, Heart, Endoplasmic reticulum stress, Apoptosis, Oxidative stress

## Abstract

Obesity and endocrine disruptors (EDs) promote endoplasmic reticulum (ER) stress, a mechanism associated with cardiovascular diseases. Among EDs, BPS is widely used in consumer products. Whether BPS promotes cardiac ER stress, either alone or combined with a high-fat diet, remains unclear. Male C57BL/6 mice were assigned to a standard chow diet (SC—15 kJ/g), SC+BPS (SCB), high-fat diet (HF—21 kJ/g), or HF+BPS (HFB), receiving BPS (25 μg/kg/day) through their drinking water for 12 weeks. Body mass (BM), lipid profile, cardiac risk ratio, heart mass, and cardiac ER stress, apoptosis, and oxidative stress markers were assessed. A high-fat diet intake increased BM gain, total cholesterol, cardiac risk ratio, and heart mass compared with standard chow diet intake. These parameters were higher in the SCB group than in the SC group. ER stress (GRP78, ATF4, and CHOP protein expression), pro-apoptotic (BAX protein expression and caspase 3 immunostaining), and pro-oxidative (NOX2 and NOX4 protein expression as well as MDA levels) markers were elevated in high-fat diet-fed animals compared with standard chow-fed animals. BPS exposure increased these parameters in standard chow-fed animals. High-fat diet intake reduced BCL2 expression and increased the BAX/BCL2 ratio compared with standard chow diet intake. BPS exposure increased BCL2 expression in standard chow-fed animals and increased the BAX/BCL2 ratio in high-fat diet-fed animals. ER stress appears to play a role in the cardiac effects of BPS. BPS exposure exacerbated the high-fat diet-induced pro-apoptotic response.

**Supplementary Information:**

The online version contains supplementary material available at 10.1007/s00418-026-02525-2.

## Introduction

The endoplasmic reticulum (ER) is an organelle that plays a crucial role in protein synthesis, folding, and transport, among other functions (Sepúlveda-Fragoso et al. 2021). Several stimuli can disturb ER homeostasis, leading to the accumulation of unfolded and misfolded proteins in a process called ER stress (Ren et al. 2021). This triggers the unfolded protein response (UPR), which aims to reestablish ER homeostasis. However, persistent UPR activation leads to apoptosis, oxidative stress, and inflammation (Sepúlveda-Fragoso et al. 2021).

ER stress has been proposed as a mechanism associated with the development of several cardiovascular diseases (CVD) (Ren et al. 2021; Sepúlveda-Fragoso et al. 2021). Among several ER stress-inducing factors related to CVD, obesity and endocrine disruptor (ED) exposure are of great interest. That obesity stresses the ER is widely known (Lee and Ozcan 2014; Petrakis et al. 2017), calling attention to the pandemic of this condition, along with current dietary patterns (Blüher 2019; Rakhra et al. 2020).

Regarding ED, bisphenol S (BPS) is of particular importance, as it is the main substitute for bisphenol A (BPA) (Brulport et al. 2021). BPA has been widely used, but several regulatory restrictions have been imposed because of its adverse health effects. Regulatory agencies worldwide have established tolerable daily exposure limits, including 50 μg/kg/day in the USA, while in the European Union, it was recently updated from 4 μg/kg/day to 0.2 ng/kg/day (Ekici and Çakır Biçer 2025; Manzoor et al. 2022). As a replacement, BPS has been broadly used in the manufacturing of food and beverage cans, toys, thermal paper, and other consumer products. However, no specific regulatory limits have been established for its use (Wu et al. 2018). Along with reproductive, metabolic, and immunological toxicity (Wu et al. 2018), BPS is associated with the development of CVD (Alexandre-Santos et al. 2024; Luo et al. 2023; Wang et al. 2022). It has been recently reported that BPS causes ER stress in the heart (Reis et al. 2026), liver (Qin et al. 2020), reproductive organs (Caglayan and Ozden 2024; Li et al. 2023), and adipose tissue (Molangiri et al. 2023).

Given the multifactorial aspect of CVD, evaluating the impact of combined factors gains significant relevance. We have previously shown that the combination of high-fat diet intake and BPS exposure aggravates cardiac hypertrophy in male mice (Alexandre-Santos et al. 2024). However, whether this response is associated with ER stress-related outcomes was not addressed by our prior work. Thus, this study aims to assess the effect of BPS exposure and/or a high-fat diet intake on cardiac ER stress, apoptosis, and oxidative stress.

## Methods

### Experimental protocol

Animal care and procedures were in accordance with the conventional guidelines for experimentation with animals (National Academy of Sciences 2011), and all protocols and procedures used in this study were approved by the Ethics Committee on Animal Use of Fluminense Federal University (protocol no. 1929240521). Animals were maintained on a 12/12-h light/dark cycle and housed in bisphenol-free polycarbonate cages in a controlled-temperature room (23 ± 2 °C; relative humidity (60 ± 5%), with food and water provided ad libitum.

Adult male C57BL/6 mice (12 weeks old) were randomly assigned to four groups (*n* = 10/group): (1) standard chow diet (SC); (2) standard chow diet + BPS exposure (SCB); (3) high-fat diet (HF); (4) high-fat diet + BPS exposure (HFB). Both diets and BPS exposure took place for 12 weeks. The diets were manufactured by PragSolucoes (São Paulo, SP, Brazil), following the recommendations of the American Institute of Nutrition (Reeves et al. 1993). The standard chow diet contained 14% protein, 10% fat, and 76% carbohydrates (15 kJ/g); the high-fat diet contained 14% protein, 50% fat, and 36% carbohydrates (21 kJ/g). BPS (103,039; 4,4′-sulfonyldiphenol) was provided by Sigma-Aldrich (St. Louis, MO, USA), dissolved in ethanol (0.1%), and added to a final concentration of 25 µg/kg/day to filtered glass-bottled drinking water. BPS concentration was adjusted weekly based on mean daily water intake and body mass (BM). SC and HF groups were exposed to an equivalent volume of ethanol (final concentration 0.1%) in their drinking water. The average BPS intake was 29.3 ± 4.40 µg/kg/day, as previously reported (Alexandre-Santos et al. 2024). The BPS dose was based on NOAEL concentrations (No Observed Adverse Effect Level) for systemic toxicity from studies using rodents as a model (https://echa.europa.eu/).

BM was measured weekly and is presented as the variation (Δ) between initial and final values.

### Tissue extraction

At the end of the 12th week, animals were deprived of food for 6 h, heparinized via intraperitoneal injection, and then deeply anesthetized with intraperitoneal ketamine (40 mg/kg) and xylazine (8 mg/kg). Blood samples were obtained by cardiac puncture, and plasma was separated by centrifugation (20 min at 120 g, at 4 ºC) and stored individually at − 80 ºC for subsequent analyses. Hearts were weighed, and left ventricles (LV) were dissected and fixed in 10% formalin (0.1 M phosphate buffer, pH 7.2) for 72 h for histological analysis or rapidly frozen and stored at − 80 °C until subsequent analysis.

### Plasma analysis

Total cholesterol and HDL levels in plasma were assessed using Bioclin® kits with a BS-120 automatic biochemical analyzer (Bioclin®, Belo Horizonte, MG, Brazil) (*n* = 10/group). The cardiac risk ratio was calculated as the ratio of total cholesterol to HDL (Mahat et al. 2018).

### Immunohistochemistry

Fixed LV samples (*n* = 5/group) were embedded in paraffin, and 5-µm-thick sections were submitted to antigen retrieval and blockade (Novolink Polymer Detection Systems Kit; Leica Biosystems, Deer Park, IL, USA), as described previously (Alexandre-Santos et al. 2019). LV sections were incubated overnight at 4 °C with primary antibody for caspase 3, followed by incubation with Envision FLEX/HRP (DAKO, 152 Agilent, SM802, Santa Clara, CA, USA) for signal amplification. Diaminobenzidine was used to detect positive immunoreaction, and hematoxylin was used for counterstaining. Negative control was performed by omitting the primary antibody. The ScanScopeTM CS (Aperio Technologies, Vista, CA, USA) was used to obtain digital images and semiquantitative scores. A score of 0 to 3 was attributed to the immunostaining intensit, and reported as: 0, no staining; 1, weak; 2, mild; 3, strong.

### Western blot

Total LV proteins were extracted in a homogenizing buffer with protease and phosphatase inhibitors (*n* = 5/group), and protein samples were resolved by SDS-PAGE 10–15% gel electrophoresis and transferred to a polyvinylidene difluoride membrane. Membranes were incubated overnight at 4 ºC with primary antibodies for CHOP, GRP78, ATF4, NADPH oxidase (NOX) 2, NOX4, Bcl-2-associated X protein (BAX), and B-cell leukemia/lymphoma-2 (BCL2). Secondary antibodies and ECL Western blot reagents were used to detect the binding of the primary antibodies, and images were acquired with the ChemiDoc system (Bio-Rad, Hercules, CA, USA). The intensity of the chemiluminescent bands was quantified using ImageJ software, version 1.44 (NIH, imagej.nih.gov/ij, Bethesda, MD, USA). Cyclophilin was used as a loading control. Detailed information for the primary antibodies is provided in Supplementary Table S1.

### Thiobarbituric acid reactive substance (TBARS) assay

Lipid peroxidation in LV homogenate (*n *= 5/group) was evaluated by measuring the level of thiobarbituric acid-reacting substances (TBARS) following the method of Ohkawa et al. (1979). Malondialdehyde (MDA) concentration was determined from absorbance at 532 nm and presented as MDA/mg of protein.

### Statistical analysis

Data are presented as means ± standard deviation (SD). Normality and homoscedasticity were tested and confirmed, and the differences among groups were verified by one-way analysis of variance (ANOVA), followed by the Holm-Sidak post hoc test. In all cases, *p* < 0.05 was considered statistically significant. All analyses were executed with GraphPad Prism (version 8.0.2, La Jolla, CA, USA).

## Results

### Bisphenol S impairs cardiac health and induces cardiac ER stress

High-fat diet intake led to higher BM gain (HF vs. SC: +364.3%, *p* < 0.0001; HFB vs. SC: +329.1%; *p* < 0.0001) and total cholesterol levels (HF vs. SC: +83.8%, *p* < 0.0001; HFB vs. SC: +67.1%, *p* < 0.0001) than standard chow diet intake (Table 1). BPS exposure increased these parameters only in standard chow diet-fed animals (BM gain: SCB vs. SC: +82.7%, *p* < 0.05; total cholesterol: SCB vs. SC: +34.2%, *p* < 0.01). HDL levels did not vary among groups. High-fat diet intake increased the cardiac risk ratio (HF vs. SC: +50.5%, *p* < 0.01; HFB vs. SC: +54.2%, *p* < 0.01) and cardiac hypertrophy, as evidenced by augmented heart mass (HF vs. SC: +14.3%, *p* < 0.05; HFB vs. SC: +16.6%, *p* < 0.05) compared with the SC group (Table 1). BPS exposure augmented both the cardiac risk ratio (SCB vs. SC: +41.5%, *p* < 0.05) and cardiac hypertrophy (SCB vs. SC: +13.2%, *p* < 0.05) only in standard chow diet-fed animals.

**Table 1:** Biometric parameters

	SC	SCB	HF	HFB
Body mass (g, Δ)	3.8 ± 1.17	7.0 ± 1.46^a^	17.7 ± 3.66^a, b^	16.3 ± 3.32^a, b^
Total cholesterol (mg/dl)	97.7 ± 12.15	131.1 ± 27.61^a^	179.5 ± 22.47^a^	163.2 ± 26.82^a^
HDL (mg/dl)	94.6 ± 15.57	90.0 ± 16.03	107.7 ± 20.59	96.1 ± 11.91
Cardiac risk ratio	1.1 ± 0.16	1.5 ± 0.29^a^	1.6 ± 0.25^a^	1.7 ± 0.44^a^
Heart mass (mg)	122.4 ± 15.17	138.5 ± 14.64^a^	139.9 ± 10.01^a^	142.7 ± 16.40^a^

Data presented as mean ± standard deviation (*n* = 10/group). Significant differences between groups are indicated with the symbols (*p*< 0.05): ^a^ ≠ SC; ^b^ ≠ SCB, as determined by one-way ANOVA and Holm-Sidak post-test. *SC* standard chow diet; *SCB* standard chow diet + BPS exposure; *HF* high-fat diet; *HFB* high-fat diet and BPS exposure

**Fig. 1:**
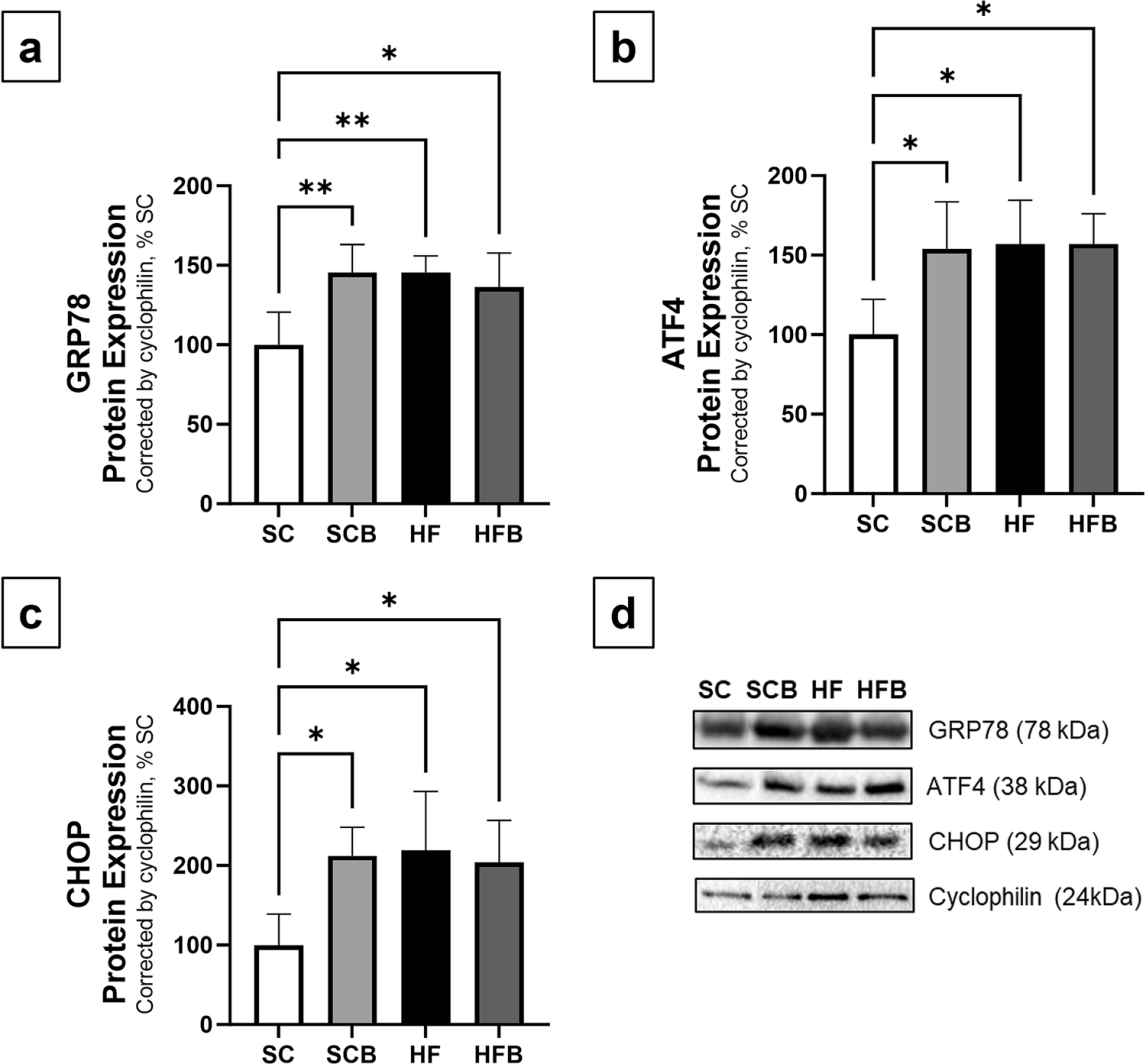
Evaluation of ER stress markers. High-fat diet intake and BPS exposure alone increased GRP78 (**A**), ATF4 (**B**), and CHOP (**C**) protein expression. Representative Western blot analysis of proteins in LV (**D**). Data presented as mean ± standard deviation (*n* = 5/group). Significant differences between groups are indicated with the symbols: *p < 0.05; **p < 0.01, as determined by one-way ANOVA and Holm-Sidak post-test. *SC* control diet; *SCB* control diet + BPS exposure; *HF* high-fat diet; *HFB* high-fat diet + BPS exposure; *GRP*78, chaperone 78 kDa glucose-regulated protein; *CHOP* C/EBP homologous protein; *ATF*4 activating transcription factor 4

Regarding ER stress, GRP78 cardiac protein expression (HF vs. SC: + 45.6%, *p* < 0.01; HFB vs. SC: + 36.4%, *p* < 0.05) was higher in high-fat diet-fed animals than in the SC group (Fig. 1A, D). Similarly, high-fat diet intake increased ATF4 (HF vs. SC: +56.9%, *p* < 0.05; HFB vs. SC: +57.1%, *p* < 0.05) and CHOP (HF vs. SC: +119.2%, *p* < 0.05; HFB vs. SC: +103.9%, *p* < 0.05) cardiac protein expression compared with the SC group (Fig. 1B–D). BPS exposure increased GRP78 (SCB vs. SC: +45.5%, *p* < 0.01), ATF4 (SCB vs. SC: +53.9%, *p* < 0.05), and CHOP (SCB vs. SC: +112.0%, *p* < 0.05) cardiac protein expression only within the standard chow diet intake.

### Bisphenol S-induced pro-apoptotic environment is counterbalanced by increased BCL2 expression

**Fig. 2:**
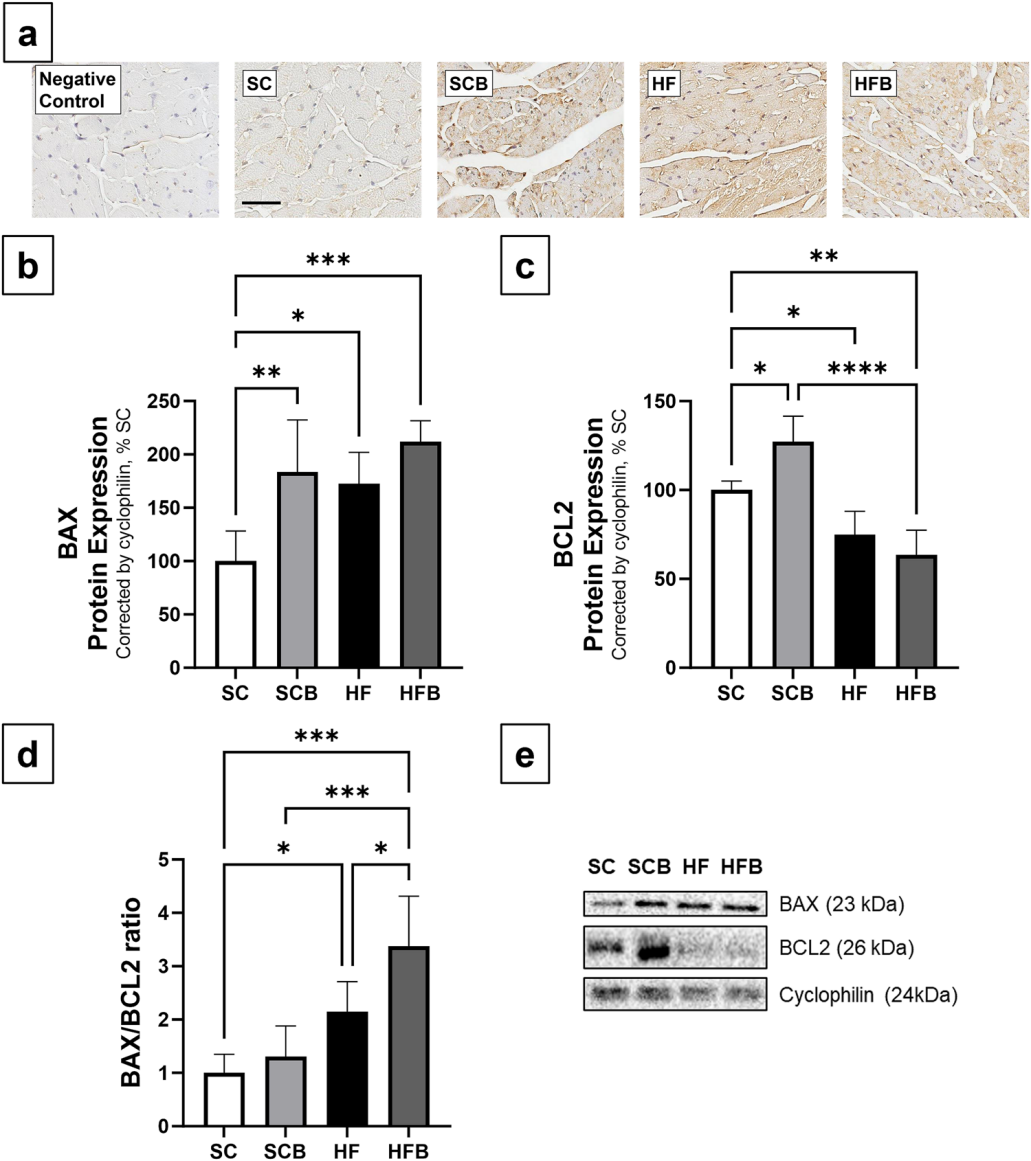
Assessment of apoptosis. Representative photomicrographs showing cardiac myofibers with strong immunoperoxidase (brown)-stained caspase 3 in the SCB, HF, and HFB groups, counterstained with hematoxylin (**A**). Same magnification in all groups (bar = 50 μm). High-fat diet intake and BPS exposure alone increased BAX (**B**) protein expression. BCL2 (**C**) protein expression was reduced after high-fat diet intake and increased after BPS exposure alone. High-fat diet intake and BPS exposure combined increased the BAX/BCL2 ratio (**D**). Representative Western blot analysis of proteins in LV (**E**). Data presented as mean ± standard deviation (*n* = 5/group). Significant differences between groups are indicated with the symbols: *p < 0.05; **p < 0.01; ***p < 0.001; ****p < 0.0001, as determined by one-way ANOVA and Holm-Sidak post-test. *SC* control diet; *SCB* control diet + BPS exposure; *HF* high-fat diet; *HFB* high-fat diet + BPS exposure; *BAX* Bcl-2-associated X protein; *BCL*2 B-cell leukemia/lymphoma-2

High-fat diet intake promoted higher BAX cardiac protein expression (HF vs. SC: +72.6%, *p* < 0.05; HFB vs. SC: +112.1%, *p* < 0.001) and stronger caspase 3 immunostaining compared with the SC group (Fig. 2A, B, E). BPS exposure increased BAX cardiac protein expression (SCB vs. SC: +83.4%, *p* < 0.01) and had stronger caspase 3 immunostaining than the SC group. The SC group had weak caspase 3 cardiac immunostaining. In high-fat diet-fed animals, BCL2 cardiac protein expression (HF vs. SC: − 25.0%, *p* < 0.05; HFB vs. SC: − 36.5%, *p* < 0.01) was lower than in the SC group (Fig. 2C, E). Interestingly, BPS exposure increased BCL2 cardiac protein expression (SCB vs. SC: 27.2%, *p* < 0.05) within standard chow diet intake. Only high-fat diet intake increased the BAX/BCL2 ratio (HF vs. SC: +114.8%, *p* < 0.05; HFB vs. SC: +237.2%, *p* < 0.001) in the SC group (Fig. 2D). In the HFB group, this ratio was exacerbated (HFB vs. HF: +37.0%, *p* < 0.05) compared with the HF group.

### Bisphenol S might increase reactive oxygen species generation and induce oxidative stress

**Fig. 3:**
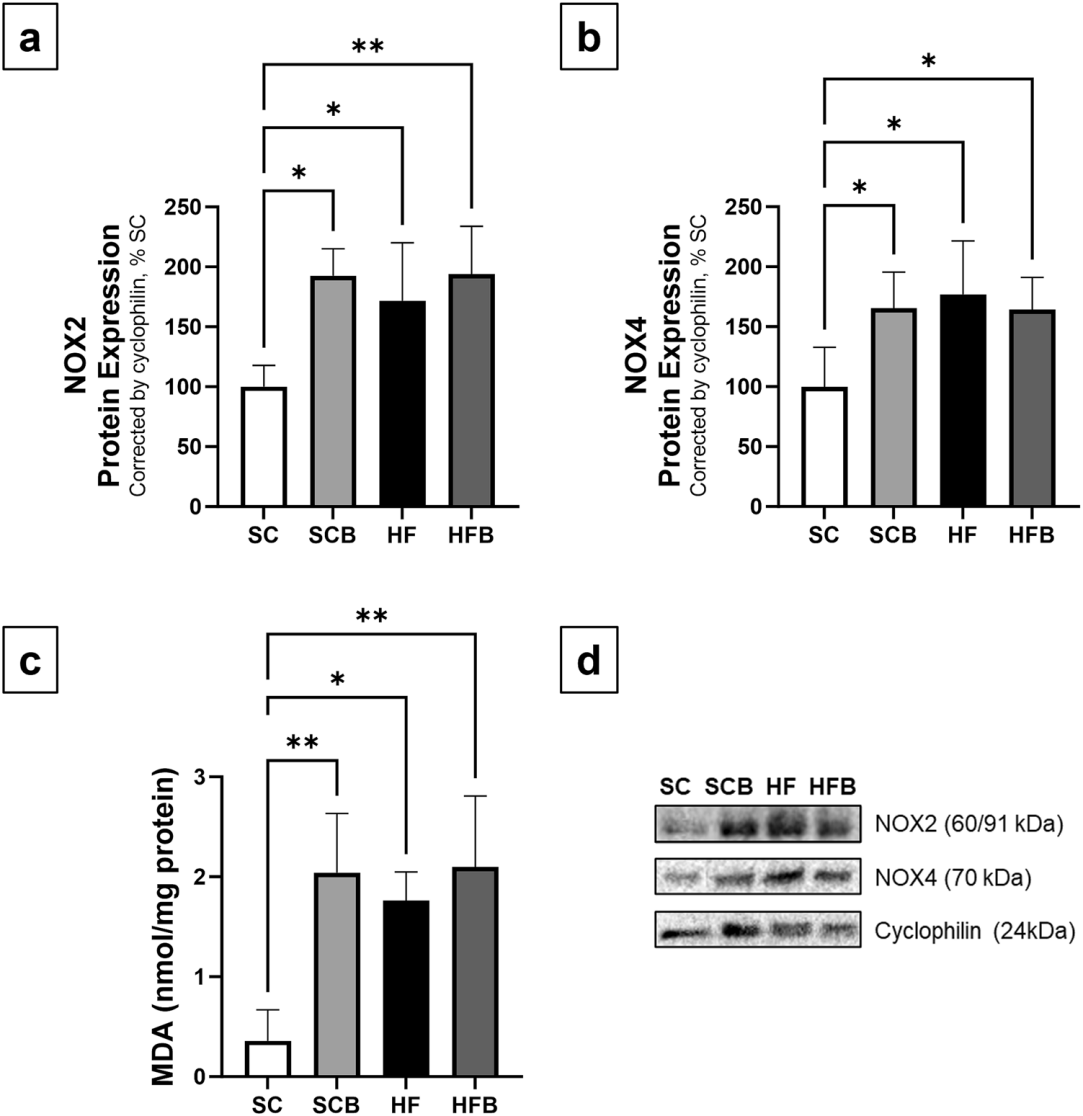
Evaluation of oxidative stress. High-fat diet intake and BPS exposure alone increased NOX2 (**A**) and NOX4 (**B**) protein expression, as well as MDA levels (**C**). Representative Western blot analysis of proteins in LV (**D**). Data presented as mean ± standard deviation (*n* = 5/group). Significant differences between groups are indicated with the symbols: *p < 0.05; **p < 0.01, as determined by one-way ANOVA and Holm-Sidak post-test. *SC* control diet; *SCB* control diet + BPS exposure; *HF* high-fat diet; *HFB* high-fat diet + BPS exposure; *NOX* NADPH oxidase; *MDA* malondialdehyde

Both NOX2 (HF vs. SC: +71.8%, *p* < 0.05; HFB vs. SC: +93.8%, *p* < 0.01) and NOX4 (HF vs. SC: +77.1%, *p* < 0.05; HFB vs. SC: +64.2%, *p* < 0.05) cardiac protein expressions were augmented in high-fat diet-fed animals compared with the SC group (Fig. 3A, B, D). Accordingly, high-fat diet intake increased lipid peroxidation (HF vs. SC: +392.3%, *p* < 0.05; HFB vs. SC: +487.4%, *p* < 0.01), which was evidenced by higher MDA levels than in the SC group (Fig. 3C). BPS exposure increased both NOX2 (SCB vs. SC: +92.6%, *p* < 0.05) and NOX4 (SCB vs. SC: +65.6%, *p* < 0.05) cardiac protein expression, as well as lipid peroxidation (SCB vs. SC: +471.2%, *p* < 0.01), within standard chow diet intake.

## Discussion

**Fig. 4:**
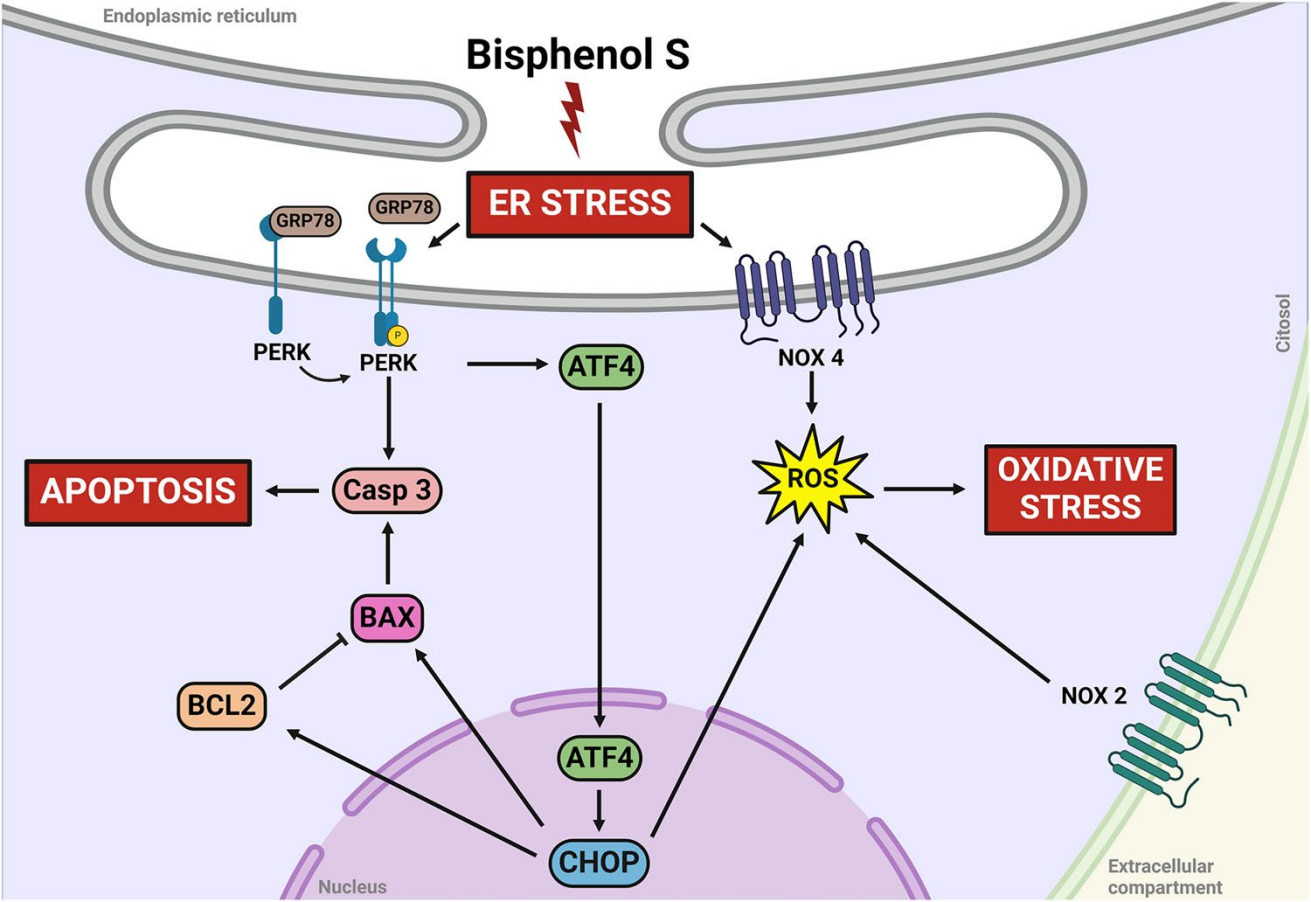
BPS induces ER stress, activating the PERK/ATF4/CHOP pathway, which leads to apoptosis and oxidative stress. PERK promotes the activation and translocation of ATF4 to the nucleus, which, in turn, induces CHOP expression. CHOP induces the expression of BAX and BCL2, activating caspase-3 and promoting apoptosis. Additionally, CHOP is responsible for increasing ROS production. Simultaneously, BPS also enhances the production of ROS via NOX2/4, intensifying oxidative stress. *ATF*4 activating transcription factor 4; *BAX* BCL2-associated X protein; *BCL2* B-cell lymphoma 2; *BPS* bisphenol S; *CHOP* C/EBP homologous protein; *ER* endoplasmic reticulum; *NOX*2/4 NADPH oxidase 2/4; *PERK* PKR-like eukaryotic initiation factor 2α kinase; *ROS* reactive oxygen species

We showed that the cardiac effects of BPS are accompanied by increased ER stress and a pro-apoptotic and pro-oxidative environment, as summarized in Fig. 4. The combination of BPS exposure and high-fat diet intake worsened the cardiac pro-apoptotic environment.

While the high-fat diet intake effects of increased BM gain, hypercholesterolemia, and cardiac hypertrophy are well described in the literature (Jin et al. 2019; Salles et al. 2024), these effects upon BPS exposure have only been recently described by us (Alexandre-Santos et al. 2024) and others (Cheng et al. 2023; Reina-Pérez et al. 2021; W. Wang et al. 2018a, b). In our experimental conditions, we have previously discussed that the combination of BPS exposure and high-fat diet intake did not act synergistically in worsening cardiometabolic parameters (Alexandre-Santos et al. 2024).

The mechanisms underlying this cardiac hypertrophic effect of BPS have been poorly explored, even though three specific sensors [PERK (PKR-like eukaryotic initiation factor 2α kinase), IRE1α (inositol-requiring protein 1α), and ATF6 (activating transcription factor-6)] mediate the UPR upon ER stress by dissociating with chaperone 78-kDa glucose-regulated protein (GRP78) (Sepúlveda-Fragoso et al. 2021). The PERK/ATF4/C/EBP homologous protein (CHOP) pathway has gained attention because of its involvement in cardiac hypertrophy, cardiomyocyte cell death, and heart failure (Ren et al. 2021; S. Wang et al. 2018a, b). ER stress is known to be involved in cardiac outcomes following high-fat diet intake (Favero et al. 2024; Salles et al. 2024; Souza-Neto et al. 2021). It has been reported that BPA induces cardiac ER stress (Liu et al. 2022), but this is only the second study to show that BPS led to ER stress and upregulated the PERK/ATF4/CHOP pathway in the heart. We have recently shown that this pathway was activated in the hearts of mice exposed to 4 and 25 µg/kg/day of BPS (Reis et al. 2026). These findings suggest that this signaling might be involved in the cardiac effects of BPS exposure. Furthermore, the association between BPS exposure and high-fat diet intake did not worsen ER stress markers, which aligns with two studies that found that BPA exposure did not aggravate high-fat diet-induced PERK pathway markers in the liver (Figueiredo et al. 2020; Kim et al. 2023).

Two of the main cellular outcomes of PERK pathway activation include apoptosis and oxidative stress (Sepúlveda-Fragoso et al. 2021). In addition to inducing CHOP overexpression, the PERK pathway also promotes caspase 12 activation (Ruan et al. 2020). Caspase 12 stimulates caspase 3, an effector caspase, culminating in cardiomyocyte apoptosis (Breckenridge et al. 2003). CHOP-induced apoptosis is mediated by BAX, a key regulator of the apoptotic response (Cosentino et al. 2022; Tabas and Ron 2011). Other processes are involved in CHOP-induced apoptosis, including downregulation of BCL2 (Tabas and Ron 2011). BAX and BCL2 work in balance to determine cell fate, and an increased BAX/BCL2 ratio indicates cell susceptibility to apoptosis, also activating caspase 3 (Gao and Wang 2009; Kale, Osterlund, and Andrews 2017). While PERK is not the only ER sensor that regulates the apoptotic program, it is one of the most well studied (Tabas and Ron 2011). High-fat diet intake has been shown to lead to cardiac apoptosis (Alrefaie et al. 2022; Park et al. 2024; Wu et al. 2022), but BPS studies are scarcer. In accordance with our findings, pro-apoptotic markers increased upon BPS exposure in cultured cardiomyocytes (Luo et al. 2023; Zhou et al. 2024). Interestingly, increased cardiac BCL2 protein expression was also described after BPS (Zhou et al. 2024) and BPA (Belcher et al. 2015) exposure, although Luo et al. observed reduced BCL2 protein expression in cultured cardiomyocytes exposed to BPS. This finding implies a cellular attempt at counterbalancing BPS-induced damage, while BAX/BCL2 ratio exacerbation in BPS-exposed high-fat diet-fed animals suggests failure in the compensatory responses, leading to a more pro-apoptotic environment. To our knowledge, this is the first study to evaluate the apoptotic response with these combined interventions, but similar results were found for BPA-exposed renal tissue (Kobroob et al. 2024).

There is an interesting interplay between ER stress and oxidative stress. While misfolded proteins themselves are a source of reactive oxygen species (ROS), CHOP mediates the expression of endoplasmic reticulum oxidoreductin 1 (ERO1) in response to activation of the PERK pathway, generating ROS (Bhattarai et al. 2021; Sepúlveda-Fragoso et al. 2021). In addition, the UPR can also activate NOXs, important cellular sources of ROS formation located on the ER membrane (Amodio et al. 2018; Gutiérrez‐cuevas et al. 2021). This increased ROS generation reinforces the cellular stress response, which might be linked to the release of proapoptotic factors, and correlates with increased lipid peroxidation (Bhattarai et al. 2021; Gianazza et al. 2021). The cardiac pro-oxidative responses of a high-fat diet are well known (Alexandre-Santos et al. 2020; Cao et al. 2011; Feriani et al. 2021). At higher doses, BPS-induced cardiac oxidative stress and lipid peroxidation have been demonstrated (Luo et al. 2023). Although we only evaluated NOXs as a potential source of ROS, our findings suggest that even a low dose of BPS dysregulates the cardiac redox balance. To our knowledge, we are the first study to show that a combination of BPS exposure and high-fat diet intake did not worsen the cardiac pro-oxidative response. This contrasts with studies in which BPA augmented hepatic and renal ROS generation in high-fat diet-fed rodents (Kobroob et al. 2024; Pirozzi et al. 2020). Even though the liver and kidneys have metabolic roles distinct from those of the heart, these studies used higher doses and different experimental conditions.

While our findings highlight an important relation between BPS exposure and high-fat diet intake on cardiac cellular outcomes, the lack of mechanistic insights reinforces the need for further studies in the field.

## Conclusion

This study reinforces the cardiac hypertrophic effect of BPS and suggests that this effect might be mediated by ER stress. Along with upregulating the PERK pathway, BPS induced a pro-apoptotic and pro-oxidative environment. The combination of BPS exposure and high-fat diet intake only exacerbated the pro-apoptotic response. Mechanistic studies are needed to fully describe BPS's impact on cardiac cellular response. These findings strengthen the need for public policies and regulation of BPS usage.

## Supplementary Information

Below is the link to the electronic supplementary material.

Supplementary file1 (PDF 89 KB)

## Data Availability

The data supporting this study’s findings are available from the corresponding author upon reasonable request.

## Abbreviations

ATF: Activating transcription factor
BAX: Bcl-2-associated X protein
BCL2: B-cell leukemia/lymphoma-2
BM: Body mass
BPA: Bisphenol A
BPS: Bisphenol S
Caspase 3: Cysteine-aspartic-acid protease
CHOP: C/EBP homologous protein
CVD: Cardiovascular diseases
ED: Endocrine disruptors
ER: Endoplasmic reticulum
ERO1: Endoplasmic reticulum oxidoreductin 1
GRP78: Chaperone 78 kDa glucose-regulated protein
IRE1α: Inositol-requiring protein 1α
LV: Left ventricle
MDA: Malondialdehyde
NOX: NADPH oxidase
PERK: PKR-like eukaryotic initiation factor 2α kinase
ROS: Reactive oxygen species
TBARS: Thiobarbituric acid-reacting substances
UPR: Unfolded protein response
