# Supplementary material for: Endoplasmic reticulum stress is associated with cardiac effects of bisphenol S independently of a high-fat diet
Source: Histochem Cell Biol. 2026 Aug 3;164(1):70. doi: 10.1007/s00418-026-02525-2 (PMC13433454; doi:10.1007/s00418-026-02525-2)
Supplement: Supplementary file 1 [file 418_2026_2525_MOESM1_ESM.pdf]

Supplementary Table 1. Primary antibodies details.

**Antibody**	**Company**	**Code**	**Datasheet**
ATF4	Santa Cruz Biotechnology, Santa Cruz, CA, USA	sc-390063	https://datasheets.scbt.com/sc-390063.pdf
BAX	Novus Biologicals, Centennial, CO, USA	NBP1-28566	https://www.novusbio.com/PDFs/NBP1-28566.pdf
BCL2	Santa Cruz Biotechnology, Santa Cruz, CA, USA	sc-7382	https://datasheets.scbt.com/sc-7382.pdf
Caspase-3	Cusabio Technology, Houston, TX, USA	CSB-PA001231	https://www.cusabio.com/datasheet/11091194/
CHOP	Santa Cruz Biotechnology, Santa Cruz, CA, USA	sc-7351	https://datasheets.scbt.com/sc-7351.pdf
GRP78	Cusabio Technology, Houston, TX, USA	CSB-PA873500	https://www.cusabio.com/datasheet/1027378/
NOX 2	Santa Cruz Biotechnology, Santa Cruz, CA, USA	sc-5826	https://datasheets.scbt.com/sc-5826.pdf
NOX 4	Santa Cruz Biotechnology, Santa Cruz, CA, USA	sc-30141	https://datasheets.scbt.com/sc-30141.pdf
Cyclophilin	Santa Cruz Biotechnology, Santa Cruz, CA, USA	sc-20361	https://datasheets.scbt.com/sc-20361.pdf

Legend: ATF4, activating transcription factor 4; BAX, Bcl-2-associated X protein; BCL2, B-cell leukemia/lymphoma-2; Caspase 3, cysteine-aspartic-acid protease; CHOP, C/EBP homologous protein; GRP78, chaperone 78 kDa glucose-regulated protein; NOX, NADPH oxidase.
